# ICDPICR2 calculates abbreviated injury scale scores from diagnostic codes: limitations for traumatic brain injury epidemiology

**DOI:** 10.1186/s40621-026-00677-2

**Published:** 2026-04-02

**Authors:** Joseph Piatt

**Affiliations:** 1 Division of Neurosurgery, Nemours Children’s Hospital Delaware, 1600 Rockland Road, Delaware, 19803 Wilmington USA; 2 Departments of Neurological Surgery and Pediatrics, Sidney Kimmel Medical College; Thomas Jefferson University, Pennsylvania, Philadelphia USA

**Keywords:** Abbreviated Injury Scale, International Classification of Diseases, ICDPICR2, Injury Severity Score, Brain Injuries, Traumatic, Software Validation

## Abstract

**Background:**

Many large data sets are limited in their usefulness for trauma epidemiology by a paucity of clinical data reflecting injury severity. Software packages have been developed for extrapolation of diagnostic codes to Injury Severity Scores commonly used in trauma research. The aim of this project was to evaluate one recently upgraded package, ICDPICR2.

**Methods:**

Data were taken from the Trauma Quality Improvement Program (TQIP) for 2022. The inclusion criterion was an Abbreviated Injury Scale (AIS) head score of 1 or greater. The ICDPICR2 package in R was used to calculate AIS body region scores and Injury Severity Scores (ISS) from the diagnostic codes in TQIP. Performance of actual and mapped scores in common epidemiological applications were compared.

**Results:**

There were 337,148 files with valid AIS head scores and known hospital discharge status. Cohen’s kappa statistics for AIS head scores and binned ISS were only fair at 0.310 and 0.280, respectively. Beyond an ISS of 15, mapped ISS underestimated actual ISS. Mapped AIS head scores and ISS had low sensitivity in identification of severe injuries, 54% and 23%, respectively. Discrimination of withdrawal of care, hospital morality and home discharge was poorer for mapped scores than for actual, and models of length of stay based on mapped scores had worse fit. Mapped scores yielded very different parameter estimates from actual scores in illustrative regression models.

**Conclusions:**

Limitations of the reliability of ICDPICR2 in administrative data sets must be anticipated. Parameter estimates from regression models constructed from mapped scores are not a reliable basis for hypothesis testing.

## Background

Abbreviated Injury Scale (AIS) scores and the composite Injury Severity Score (ISS) are used widely in clinical trauma research and epidemiology. For each of the 9 body regions there is a list of dozens of descriptions of possible injuries. Each description is assigned an identifying 6-digit “PREDOT” code and a single-digit severity code between 1 and 6. An injury of severity 6 is expected to be fatal. If the trained reviewer is unable to assign an injury description after examination of the entire medial record, a score of 9 is applied. An ISS is then calculated as the sum of the squares of the three maximum body region scores.

At trauma centers verified by the American College of Surgeons, these registrar-assigned scores are recorded in institutional registries and then submitted for inclusion in the National Trauma Data Base (NTDB) and the Trauma Quality Improvement Program (TQIP) registry. The NTDB and TQIP have played large roles in trauma research, but they have limitations: They reflect care only at organized trauma centers, and they are not population-based. They do not support nationwide or regional estimations of the incidence of injuries.

On the other hand, the Healthcare Cost and Utilization Project of the Agency for Healthcare Research and Quality compiles a family of data sets based on a complex survey methodology that supports estimations of population-based incidences. These data sets, which are publicly available on request for modest cost, would be an indispensable resource for trauma epidemiology but that they lack clinical detail. Specifically, they lack descriptors of severity of injury such as physiological data, Glasgow Coma Scale scores, or AIS scores and ISS. Other large data sets from Medicare, Medicaid, the Pediatric Health Information System, and commercial claims data share these deficiencies to varying degrees. A reliable algorithm for translating diagnostic codes into AIS scores and ISS would thus unlock vast quantities of data for trauma research.

ICDPICR is a statistical software package developed for this purpose. Originally developed in STATA, it has been popularized as a package in R [1]. It has undergone validation studies based on institutional registries, the NTDB, and TQIP with variable results [1–9]. Although the output of ICDPICR includes AIS scores for individual body regions, New Injury Severity Scores, extrapolations of mechanisms and intents of injury, and several models of mortality, attention has focused on its mappings of ISS, which have been found generally to underestimate registrar-assigned scores [1–5, 7]. The popularity of ICDPICR in published trauma research is difficult to assess, as it may not be mentioned in the Methods sections of abstracts, but it appears to have found some acceptance [10–14]. 

The aim of the current work is to evaluate the newest version of the software in R, ICDPICR2, in the context of analyses that might arise in traumatic brain injury (TBI) epidemiology [15]. The consistency of actual and mapped scores is assessed, discrimination of various outcomes is compared, and the validity of hypothesis tests based on parameter estimates is examined in multivariate regression models incorporating actual and mapped scores.

## Methods

Data were taken from the Participant User Files (PUF) of the Trauma Quality Improvement Program (TQIP) registry for 2022. The study sample consisted of all files with an AIS head score of 1 or greater and a known survival status at discharge. Cases transferred to another acute care facility and cases with hospital disposition data missing were excluded. For many individual cases more than one AIS head score was recorded. For each case the maximal AIS head score was carried forward for analysis. For the purposes of this study, the registrar-assigned AIS scores and ISS were ground truth.

The ICDPICR2 package for the R statistical software system was downloaded from the Comprehensive R Archive Network repository [15]. This package accepts as input ICD diagnostic codes – as many as 50 for each TQIP file - and estimates AIS scores for all 6 body regions. The ICDPICR2 package assigns AIS scores only from 0 to 5. It does not assign AIS scores of 6 or 9.

AIS head scores, both mapped and actual, were analyzed as categorical data. As cases with AIS head scores of 0 were excluded from the study sample, a score of 1 was taken as the regression reference for both. An actual AIS head of 9 was considered missing. ISS, both mapped and actual, were binned for categorical analysis as 1 to 8, 9 to 15, 16 to 24, 25 to 40, 41 to 49, and 50 to 70. In regressions mapped and actual ISS were analyzed as numerical data with non-linear effects, including quadratic and cubic terms. Age was binned as 0 to 5, 6 to 12, 13 to 17, 18 to 60, and > 60 years, and it was analyzed with adults, 18 to 60, as the reference. Outcomes were “mortality,” including discharge to hospice, “withdrawal of care,” and “home,” including discharge with home nursing, discharge against medical advice, discharge to law enforcement, and discharge to a psychiatric institution. Complete case analyses were performed.

The performances of mapped and actual, registrar-assigned scores were compared in a variety of analyses that might arise in an epidemiological study. Mapped scores were compared directly with actual scores by confusion matrices, by a Bland-Altman plot, and by the Cohen’s kappa statistic. Sensitivity, specificity, positive and negative predictive values of mapped scores were presented for certain severity thresholds with confidence intervals (CIs). Discriminations of logistic regression models based on actual and mapped scores were compared by areas under the curve (AUC) and the DeLong test. Pairs of logistical regressions were constructed based on mapped and actual scores, and differences in parameter estimates and confidence intervals were analyzed. Length of stay was modeled using ordinary least squares (OLS) based on actual and mapped ISS. Because of the skew of length of stay, confidence intervals were bootstrapped with 1000 samplings.

Data were organized and analyzed in R Studio [16]. In addition to the ‘ICDPICR2’ package already mentioned, the “psych” package was used to calculate Cohen’s kappa and the “pROC” package was utilized to assess model discrimination [17, 18]. The p-value “0” is used to designate *p* < 2.2 × 10^− 16^, which is the smallest p-value estimated by the software package.

The Committee on Trauma of the American College of Surgeons requires the following statement:

TQP PUF Version Admissions Year.2022, Chicago, IL, 2022 The content reproduced from the PUF remains the full and exclusive copyrighted property of the American College of Surgeons. The American College of Surgeons is not responsible for any claims arising from works based on the original data, text, tables, or figures.

## Results

There were 1,232,956 cases in the 2022 TQIP Participant User Files. 409,187 cases had valid head AIS scores. Discharge dispositions were known in 337,148 cases, and these cases constituted the study sample. The mortality rate was 8.9%. The rate of home discharge was 66.3%. Support was known to have been withdrawn in 4.4% of cases.

### AIS head scores

Actual and mapped head AIS scores are presented in a confusion matrix (Table 1).

**Table 1:** A confusion matrix of actual and mapped AIS head scores

		Mapped					
		0	1	2	3	4	5
	1	507	**74**,**043**	7,822	5,980	1,603	93
	2	425	36,246	**22**,**558**	19,252	2,368	63
	3	2,033	8,721	8,513	**30**,**559**	32,418	213
Actual	4	276	1,397	2,057	14,673	**21**,**413**	97
	5	314	1,022	955	8,734	19,449	**2**,**436**
	6	2	6	6	12	33	107
	9	26	713	8,209	800	629	62

Scores in agreement are bolded

Because ICDPICR2 did not map any cases onto AIS scores of 6 or 9, a degree of disagreement is a feature of the package. In this data set, however, actual AIS head scores of 6 or 9 accounted for only 3.1% of cases, so the effect of this structural disagreement was small. ICDPICR2 judged that 3,583 cases (1.1%) had not had a head injury at all. It assigned mapped head AIS scores to 10,439 cases (3.1%) for which registrars were unable to assign a score. Mapped and actual scores agreed in only 151,008 cases (45%). Cohen’s kappa statistic for scores 1 through 5 (where agreement was possible) was fair at 0.310. A plurality of actual AIS 2 scores were mapped at 1. A plurality of actual AIS 3 scores were mapped at 4, as were a plurality of actual AIS 5 scores. AIS head scores of 4, 5, and 6 are commonly considered severe head injuries. The sensitivity of mapped AIS head scores in identifying severe head injury was only 0.538 (95% CI 0.534–0.541). The specificity was 0.843 (95% CI 0.842–0.845). The positive predictive value was 0.530 (95% CI 0.527–0.534). The negative predictive value was 0.847 (95% CI 0.846–0.849).

### ISS

**Fig. 1:**
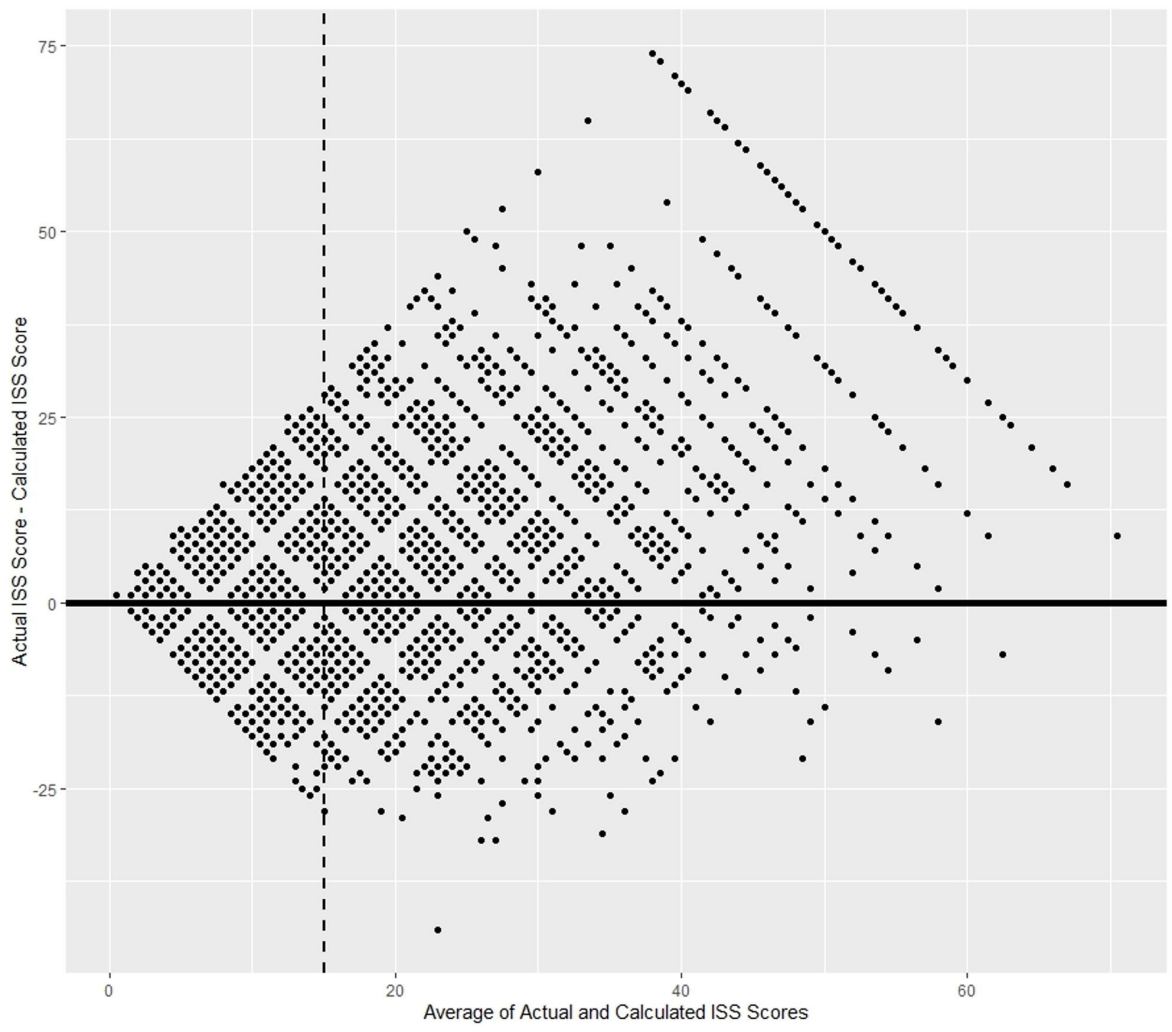
A Bland-Altman plot comparing actual and estimated ISS. Beyond the average ISS threshold of 15, marked by a vertical dashed line, mapped scores markedly underestimated actual scores

The distributions of actual and mapped ISS were highly significantly different by the Wilcoxon test (p = “0”). Actual ISS ranged from 1 to 75. Mapped ISS ranged from 0 to 66. The median actual ISS score was 10 [5–17], while the median mapped ISS score was 9 [4–16]. A Bland-Altman plot of actual and mapped ISS appears in Fig. 1. Beyond an average score of 15, mapped ISS underestimated actual scores. A confusion matrix of binned scores is presented in Table 2.

**Table 2:** A confusion matrix of actual and mapped ISS

		Mapped					
		1–8	9–15	16–24	25–40	41–49	50–70
	1–8	**80**,**798**	17,625	1,659	69	1	0
	9–15	43,242	**44**,**960**	28,595	1,045	5	0
	16–24	9,124	25,861	**27**,**534**	3,322	31	3
Actual	25–40	2,769	11,376	24,178	**7**,**778**	256	17
	41–49	55	224	1,161	1,715	**231**	30
	50–70	34	79	461	1,439	353	**86**
	NA	977	40	13	2	0	0

Scores in agreement are bolded; NA = missing data

Cohen’s kappa statistic was fair at 0.280. ISS *≥* 25 is a common definition of severe injury. Mapped ISS had a sensitivity of only 0.228 (95% CI 0.224–0.231) in detection of severe injury. The specificity was 0.984 (95% CI 0.984–0.985). The positive predictive value was 0.727 (95% CI 0.720–0.736). The negative predictive value was 0.874 (95% CI 0.873–0.875).

Associations of actual and mapped ISS with various outcomes were modeled by logistic regression. As the associations were non-linear in ISS, optimal models included quadratic and cubic terms. The models are compared in Table 3.

Actual ISS had better discrimination for all outcomes, but the discrimination differences between actual and mapped were greatest for the least frequent outcomes.

**Table 3:** Discriminations of actual and estimated ISS for various outcomes

Outcome	Incidences	Actual ISS (AUC)	Mapped ISS (AUC)	*p* - value
Mortality	29,882/337,148 (8.9%)	0.7753	0.7476	“0”
Withdrawal of care	14,007/316,836 (4.4%)	0.7982	0.6740	“0”
Home	223,393/337,148 (66.3%)	0.6860	0.6740	“0”

AUC = area under the curve; “0” signifies *p* < 2.2 × 10^− 16^, which the smallest p – value estimated by the software package

### Performance in logistic regression

To illustrate the performances of actual and mapped scores in logistic regression, a basic regression of mortality against AIS head scores, ISS, and age groups was performed (Table 4).

**Table 4:** Mortality regressed against ISS, AIS head scores, and age groups for actual and mapped scores

Covariates	Levels	Counts	Actual scores Odds ratios (95% CI)	Estimated scores Odds ratios (95% CI)
ISS				
	ISS		**1.04 (1.03–1.05)**	**1.05 (1.04–1.06)**
	ISS^2		**1.00 (1.00–1.00)**	**1.00 (1.00–1.00)**
	ISS^3		**1.00 (1.00–1.00)**	**1.00 (1.00–1.00)**
AIS head				
	0		none	**3.19 (2.74–3.70)**
	1		ref	ref
	2**		**0.80 (0.75–0.85)**	**1.44 (1.34–1.53)**
	3**		**1.37 (1.30–1.45)**	**1.96 (1.85–2.08)**
	4**		**1.76 (1.66–1.88)**	**3.33 (3.12–3.55)**
	5**		**5.59 (5.25–5.95)**	**10.7 (9.66–11.8)**
	6		**13.9 (8.57–23.4)**	none
Age group				
	0–5*	6,560	**0.59 (0.50–0.68)**	**0.73 (0.63–0.84)**
	6–12*	6,883	**0.41 (0.35–0.49)**	**0.52 (0.44–0.61)**
	13–17**	11,288	**0.68 (0.61–0.75)**	**0.84 (0.76–0.93)**
	18–60	137,638	ref	ref
	> 60 yrs**	148,208	**2.49 (2.41–2.57)**	**2.16 (2.09–2.22)**

A single asterisk (*) signifies that each parameter estimate lies outside the confidence interval of the other. A double asterisk (**) signifies non-overlapping parameter estimate confidence intervals. Parameter estimates and confidence intervals in bold are significantly different from reference levels

The AUC of the model based on actual scores was 0.8425, and for the mapped scores it was 0.7952 (p = “0”). Note that the confidence intervals for the odds ratios of the AIS head scores in the two models were non-overlapping at each level, an expected finding in view of the poor agreement between actual and mapped AIS scores. The odds ratios for the age groups were very different between the models as well, and the confidence intervals for teenagers and the elderly were non-overlapping. Regression with mapped scores can, however, provide a valid reflection of some qualitative relationships. For example, the odds ratios for AIS head scores increased monotonically in the regression on mapped scores as in the regression on actual scores. Likewise, the odds ratios for the elderly age group were the largest, and the odds ratios for the 6 to 12 age group were the smallest in both regressions.

To the basic model of Table 4 were added one at a time single covariates reflecting race, ethnicity, and insurance payer. (Insurance payer was added as an interaction with age group, in view of the different implications of non-commercial insurance in children and in the elderly.) The odds ratios and confidence intervals for the various levels of each of these covariates added individually to the basic model are presented in Table 5.

**Table 5:** Associations of race, ethnicity, and insurance payer added individually to the basic model of mortality regressed against ISS, AIS head scores, and age groups

Covariates	Levels	Counts	Actual scores Odds ratios (95% CI)	Mapped scores Odds ratios (95% CI)
Race				
	White	245,974	ref	ref
	Asian***	10,182	**0.78 (0.72–0.85)**	1.01 (0.93–1.09)
	Black	40,411	0.98 (0.94–1.03)	1.00 (0.95–1.04)
	Other	32,554	**0.87 (0.82–0.92)**	**0.89 (0.85–0.94)**
	Unknown	8,027	**1.69 (1.56–1.83)**	**1.76 (1.63–1.89)**
Ethnicity				
	Non-Hispanic White	272,807	ref	ref
	Hispanic	41,467	**0.88 (0.84–0.92)**	**0.90 (0.86–0.94)**
	Unknown	12,435	**1.45 (1.36–1.55)**	**1.49 (1.40–1.59)**
Insurance				
	Commercial	118,931	ref	ref
	Other	211,680	**1.51 (1.43–1.58)**	**1.49 (1.42–1.56)**
Age group ^x^ Insurance				
	0–5 ^x^ other	3,625	1.02 (0.73–1.45)	1.03 (0.75–1.43)
	6–12 ^x^ other	3,297	0.92 (0.65–1.31)	0.89 (0.64–1.23)
	13–17 ^x^ other	5,007	**1.34 (1.09–1.65)**	**1.24 (1.02–1.51)**
	> 60 yrs ^x^ other	113,412	**0.88 (0.83–0.95)**	**0.93 (0.87–0.99)**

A superscript ”x” (^x^) indicates an interaction in the regression model. A triple asterisk (***) signifies conflicting conclusions about statistical significance. Parameter estimates and confidence intervals in bold are significantly different from reference levels

Notably, Asian race had a highly significant protective effect in the model based on actual scores, while in the model based on mapped scores, Asian race was not associated with mortality. The confidence intervals were non-overlapping. The protective effect of Asian race persisted when it was added to the basic model as a binary covariate (data not shown).

### Performance in ordinary least squares regression

Length of stay was regressed against actual and mapped ISS using OLS. Race was added to this model for illustration. Parameter estimates and confidence intervals are presented in Table 6.

**Table 6:** Ordinary least squares regression of length of stay against ISS and race

Covariate	Actual ISS	Mapped ISS
Intercept**	**2.358 (2.292–2.421)**	**2.946 (2.890–3.002)**
ISS**	**0.371 (0.366–0.377)**	**0.409 (0.403–0.415)**
White	ref	ref
Asian** ***	0.938 (0.750–1.117)	**1.754 (1.549–1.961)**
Black**	**1.344 (1.228–1.464)**	**1.616 (1.509–1.718)**
Other*	**0.474 (0.356–0.579)**	**0.699 (0.579–0.813)**
Unknown*	**0.278 (0.023–0.536)**	**0.670 (0.423–0.924)**
Residual Standard Error	10.44	10.61

A single asterisk (*) signifies that each parameter estimate lies outside the confidence interval of the corresponding other. A double asterisk (**) signifies non-overlapping parameter estimate confidence intervals. A triple asterisk (***) signifies conflicting conclusions about statistical significance. Parameter estimates and confidence intervals in bold are significantly different from zero or from reference levels

Parameter estimates were consistently larger in the model based on mapped ISS. Once again the model based on actual scores and the model based on mapped scores came to differing conclusions about the significance of the association of Asian race with the outcome, length of stay. The residual standard error of the regression with actual ISS was significantly lower than with mapped ISS (mean difference − 0.165, bootstrapped 95% CI -0.176 to -0.154).

## Discussion

ICDPICR2 maps diagnostic codes into AIS scores and thence into ISS. As originally conceived and as utilized in contemporary trauma registries, AIS scores are hand-made. That it, they are the product of human registrars reviewing medical records. Although strenuous efforts are made, there is no expectation of perfect accuracy or perfect inter-rater reliability. A software package that outputs AIS scores purports to match hand-made AIS scores, so the hand-made scores are ground truth. Validation of ICDPICR2 must therefore compare mapped and hand-made, actual scores directly, but it can also compare mapped and actual scores as predictors of real-world outcomes.

The original versions of ICDPICR in R and in STATA have been validated in a limited fashion in various data sets. Di Bartolomeo et al. studied the STATA version in an Italian trauma registry coded in ICD-9-CM [5]. They noted that the discrepancies between actual and mapped ISS increased with the number of AIS scores in each case file. Discrimination of mortality was lower for mapped ISS. Green et al. studied the STATA version in an American regional trauma registry [6]. AIS and ISS mapped by ICDPICR were closer to actual scores than the ICD-9-CM embedded severity codes. Sears et al. used ICDPICR and another STATA package, ICDMAP, to predict time lost and total disability based on ICD-9-CM codes in a Washington State workers’ compensation registry [19]. Discrimination of mortality was the focus of the validation study of Van Belleghem et al., again on the basis of ICD-9-CM codes [20]. In a cooperative study from 9 American trauma registries, Fleischman et al. compared actual ISS with scores mapped by ICDMAP and ICDPIC [2]. Mapped scores underestimated actual scores. The developers of the first R package, Clarke et al., published a validation study based on comparisons with scores mapped by the STATA package and by discrimination of mortality [1]. 

So far as the author is aware, ICDPICR2 has not been subjected to validation study. Like previous work the current study compared mappings of AIS head scores and ISS to the ground truth of certified trauma registrar-assigned scores. In the restricted range where agreement between actual and mapped AIS head scores could be assessed, it was only fair by Cohen’s kappa statistic. Mapped head scores tended to be lower than actual scores, and as a metric for identifying severe injury (AIS head 4, 5, or 6), mapped scores were seriously deficient with a sensitivity of only 54%. Likewise, agreement between mapped and actual binned ISS was only fair by the kappa statistic, and beyond an ISS of 15 mapped scores underestimated actual scores. The sensitivity of mapped ISS to severe injury (actual ISS *≥* 25) was only 23%. Discrimination of real world outcomes by models based on mapped ISS was consistently poorer than in models based on actual scores. The OLS model of length of stay based on actual scores had better fit than the model based on mapped scores.

Beyond the scope of past work, the current study examined the performance of mapped scores in multivariate modeling. Qualitative patterns of associations were similar to models with actual scores, but individual parameter estimates were often very different. Parameter estimates from models with actual scores and from models with mapped scores often lay outside each other’s confidence intervals. Such discrepancies are a substrate for differing conclusions in hypothesis testing. For example, if the research question were whether the risk of death for Asian victims is the same as for White victims, the investigator would draw the wrong conclusion from mapped AIS head scores and mapped ISS (Table 5). Likewise if the question were equality of lengths of stay (Table 6). Differences between models incorporating actual and mapped scores seemed most pronounced in analysis of less frequent outcomes and of covariate categories of very low prevalence. In OLS modeling parameter estimates using mapped ISS were consistently greater than parameter estimates based on actual ISS, possibly because of the underestimation of actual ISS by mapped scores beyond the threshold of 15. Parameter estimates from models constructed from mapped scores are not a reliable basis for hypothesis testing.

### Limitations

The ICD-10-CM diagnostic codes that served as input for the ICDPICR2 calculations were taken from TQIP, a dedicated trauma data set, and they have been edited retrospectively to harmonize with the registrar-assigned AIS scores. But ICDPICR2 was not intended for use in trauma data sets. It was intended for use with diagnostic codes assigned by general purpose coders working in the health information departments of hospitals that may or may not provide substantial volumes of trauma care. ICDPICR2 cannot be expected to perform better in such an environment than in TQIP.

Because of the author’s interests and because of the prominence of AIS head scores in trauma research, this work has focused on the head to the neglect of other body regions. Observations about the deviation of mapped AIS head scores from actual scores ought not to be translated uncritically to those other regions. Observations about mapped ISS are not subject to this caveat.

### Conclusions

Here is reported the first analysis of the validity of the updated ICDPICR2 package for estimation of AIS head scores and ISS from ICD-10-CM diagnostic codes. Agreement between actual and mapped AIS head scores and ISS was only fair, and mapped scores underestimated severity in the more severe ranges. Models based on actual scores had closer conformity to reality than models based on mapped scores. In multivariate modeling parameter estimates based on actual and mapped scores often lay outside each other’s confidence intervals, creating a substrate for differing conclusions about statistical significance. An individual parameter estimate modeled on mapped scores cannot be a reliable basis for hypothesis testing. Limitations of the reliability of ICDPICR2 in administrative data sets must be anticipated.

## Data Availability

The data set analyzed in the current study, the 2022 TQIP Participant Use File, is available from the American College of Surgeons. The data use agreement under which the current study was performed precludes sharing of these data without permission from the American College of Surgeons.

## Abbreviations

ACS: American College of Surgeons
AIS: Abbreviated Injury Scale
AUC: Area under curve
ICD-9-CM: International Classification of Disease, 9th Edition, Clinical Modification
ICD-10-CM: International Classification of Disease, 10th Edition, Clinical Modification
ISS: Injury Severity Scale
NTDB: National Trauma Data Bank
PUF: Participant Use File
TBI: Traumatic brain injury
TQIP: Trauma Quality Improvement Program
TQP: Trauma Quality Programs
